# eHealth literacy and socioeconomic and demographic characteristics of parents of children needing paediatric surgery in Sweden

**DOI:** 10.1002/nop2.1316

**Published:** 2022-08-25

**Authors:** Ólöf Kristjánsdóttir, Anna Welander Tärneberg, Pernilla Stenström, Charlotte Castor, Inger Kristensson Hallström

**Affiliations:** ^1^ Faculty of Nursing University of Iceland Reykjavík Iceland; ^2^ Department of Economic History, Centre for Economic Demography Lund University Lund Sweden; ^3^ Department of Pediatric Surgery and Neonatology Skåne University Hospital, Lund University Lund Sweden; ^4^ Department of Health Sciences, Lunds Universitet Lund Sweden

**Keywords:** caregiver, digital intervention, eHealth, eHealth literacy, health equity, paediatric surgery, paediatrics, parent, socioeconomic status

## Abstract

**Aim:**

The aim of the study was to describe different eHealth literacy domains among parents of children needing paediatric surgery in Sweden, and the correlation between these eHealth literacy domains and parents' socioeconomic factors and demographic characteristics.

**Design:**

Descriptive correlational design.

**Method:**

Thirty‐five Swedish‐speaking parents participated as a historical control group within an ongoing Swedish clinical trial developing eHealth solutions for families after hospital care; of these, 30 completed the eHealth Literacy Questionnaire and the socioeconomic and demographic questionnaire.

**Results:**

Of the seven eHealth literacy domains assessed, parents' strengths lay in those pertaining to their own digital competence, control and safety, while their weakness concerned their motivation to engage with digital services, and their ability to access eHealth platforms that work. Overall, parents presented adequate eHealth literacy. Of the five socioeconomic and demographic variables assessed (i.e. monthly wages, education levels, age, gender and residency), monthly wages correlated the strongest, and positively, with the seven eHealth literacy domains.

## 1 INTRODUCTION

The demand for and interest in electronic health (eHealth) services, with eHealth simply referring to “the transfer of health resources and healthcare by electronic means” (WHO Regional Office for Europe, 2014), has accelerated during the COVID‐19 pandemic and beyond (Wong et al., 2021). This has created an urgency for people to be electronically health literate (eHealth literate) (Brørs et al., 2020).

eHealth literacy is a relatively new concept referring to “the ability to identify and define a health problem, to communicate, seek, understand, appraise and apply eHealth information and welfare technologies in the cultural, social and situational frame and to use the knowledge critically in order to solve the health problem” (Gilstad, 2014, p. 69). In a digital context, individuals need adequate eHealth literacy to fully benefit from eHealth participation. Inability to do so can fuel inequity, where individuals with equal needs do not have equal access to health care (Boeckxstaens et al., 2011; Cheng, Beauchamp, et al., 2020). As one of the Nordic countries seeking to ensure health equity, Sweden has put into place the Health and Medical Services Act (Hälso‐ och Sjukvårdslag, SFS 2017:30), stating that everybody has the right to receive care according to their needs. Thus, factors such as gender, socioeconomic status or residency should not influence access to health services and healthcare efforts. To push this effort, Sweden has advocated the eHealth platform to promote good and equal health care for its citizens (Swedish Government and Swedish Association of Local Authorities and Regions [SALAR], 2020).

The digitalization of health care has raised concerns about the development of effective and equitable eHealth interventions, particularly across different socioeconomic positions (El Benny et al., 2021). To promote equity and effectiveness in digital intervention planning and implementation, scientists have started to suggest assessing different domains of eHealth literacy. Such assessment is believed to create a unique understanding of end‐users' attributes, that is a profile of their strengths and weaknesses in specific domains of eHealth literacy, as well the responsivity of the digital services (Cheng, Beauchamp, et al., 2020; El Benny et al., 2021; Kayser et al., 2018).

Parents of children with serious or long‐term illnesses are a vulnerable group; overburdened, and at a higher risk of developing health problems (Graj et al., 2021; Raina et al., 2005), but with digitalization the expectation of parental involvement and self‐management continues to increase in paediatric care (Barros & Greffin, 2017; Bird et al., 2019). Importantly, these parents report digital communication as an attractive, effective and safe way to communicate with healthcare professionals (Goedeke et al., 2019; Lindkvist et al., 2021). However, the extent to which these parents can effectively use and fully benefit from digital health services remains unclear.

Our understanding of eHealth literacy among parents in general, and in paediatrics, is therefore warranted. The existing literature originates from the United States (Knapp, Madden, Marcu, et al., 2011; Knapp, Madden, Wang, et al., 2011), Australia (Kasparian et al., 2017) and Switzerland (Juvalta et al., 2020), using samples from clinical (Kasparian et al., 2017; Knapp, Madden, Marcu, et al., 2011; Knapp, Madden, Wang, et al., 2011) and non‐clinical (Juvalta et al., 2020) settings, always using the eHealth Literacy Scale (eHEALS) (Norman & Skinner, 2006). Of particular concern, however, is that these studies suggest that a considerable eHealth literacy gap exists, particularly in parents' awareness of, access to, and communication about internet‐based health information. Furthermore, to the best of our knowledge, the eHealth literacy of parents of children needing paediatric surgery has not been studied outside nor within Nordic contexts.

eHealth literacy and socioeconomic conditions are reported to be related constructs, and individuals with lower socioeconomic status have shown lower eHealth literacy (Chesser et al., 2016; Karnoe & Kayser, 2015; Kim & Jeon, 2020). In studies of vulnerable populations (e.g. patients, caregivers, older adults, immigrants and low‐income minority groups) less capabilities, access and experience in using digital health services and technology have been shown (Arsenijevic et al., 2020; Azzopardi‐Muscat & Sørensen, 2019; Meyers et al., 2020; Thorsen et al., 2020). This trend has also been seen in studies of parents of children with special needs (Knapp, Madden, Wang, et al., 2011) and those of children with critical illnesses (Kasparian et al., 2017; Knapp, Madden, Marcu, et al., 2011).

Limited data exits on the eHealth literacy of parents with children in paediatric care in Sweden, and there are no reports at all from paediatric surgery. To promote and ensure health equity, exploring parents' eHealth literacy within a socioeconomic context is recommended. Therefore, the main aim of the present study was to explore different eHealth literacy domains of parents with children needing paediatric surgery in Sweden. The secondary aim was to explore the correlation between different eHealth literacy domains and parents' socioeconomic and demographic characteristics.

## 2 METHODS

### 2.1 Design

A quantitative descriptive correlational approach was applied based on data collection for a historical control group in an ongoing Swedish experimental controlled clinical trial developing eHealth solutions for families after hospital care (ClinicalTrials.gov identifier: NCT04150120). Furthermore, the current study is part of and conducted within the larger context of an international research initiative—eChildHealth—led by Lund University in Sweden and centred around health equity and user participation, with the objective of creating eHealth solutions in both high‐ and low‐income countries that support self‐management in families of children needing advanced paediatric care (Hallström et al., 2018).

**FIGURE 1:**
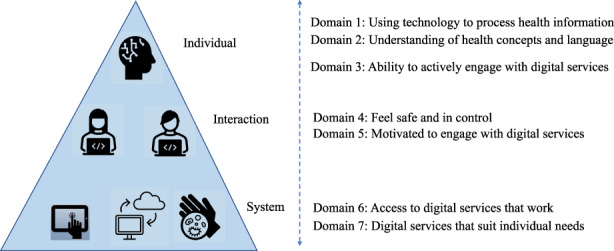
Seven dimensions of the eHealth literacy framework (eHLF) as presented by Norgaard et al. (2015) and Kayser et al. (2018)

Methodologically, this study builds on the seven‐domain eHealth Literacy Framework (eHLF) (Kayser et al., 2015, 2018), shown in Figure 1, illustrating the conceptualization of eHealth literacy as the attributes of the individual, context or interaction, and system. The underpinnings of eHLF are considered a useful set of tools to support and create eHealth solutions facilitating equity, accessibility and sustainability (Azzopardi‐Muscat & Sørensen, 2019; Cheng, Beauchamp, et al., 2020).

### 2.2 Participants, setting and data collection

The study was conducted over a 6‐month period from February to July 2020 at a tertiary referral centre of paediatric surgery at a university hospital in the south of Sweden. Participants were included based on convenience sampling, including legal guardians (parents) aged ≥18 years old who were able to read and write Swedish and with a child below 4 years of age undergoing paediatric surgery. A total of 35 answered an online survey; of these, 30 completed the questionnaires analysed in this study.

### 2.3 Measures

#### 2.3.1 eHealth literacy

The eHealth Literacy Questionnaire (eHLQ) (Kayser et al., 2018) was used to measure eHealth literacy, but this tool was created from and designed to capture the eHLF (Figure 1). This self‐report measure evaluates people's interaction with digital health services and technology in relation to their health. Validity testing of the eHLQ has shown satisfactory evidence of construct validity and reliability across various settings (Kayser et al., 2018). The eHLQ comprises 35 items in the following seven independent domains. eHLQ1: Using technology to process health information (5 items), eHLQ2: Understanding of health concepts and language (5 items), eHLQ3: Ability to actively engage with digital services (5 items), eHLQ4: Feel safe and in control (5 items), eHLQ5: Motivated to engage with digital services (5 items), eHLQ6: Access to digital services that work (6 items) and eHLQ7: Digital services that suit individual needs (4 items). Dimensions eHLQ1–3 describe parents' competence (i.e. the individual), dimensions eHLQ4–5 describe the interaction between the parent and the digital services (i.e. the context) and dimensions eHLQ6–7 describe parents' experience with digital health services (i.e. the system) (Figure 1). Each item is scored using 4‐point ordinal scale options: 1 (strongly disagree), 2 (disagree), 3 (agree) and 4 (strongly agree). Appendix A displays the 35 items within the seven eHLQ domains or scales.

For each of the seven eHLQ domains, total scores are calculated by averaging the item scores within each scale, each with a score range of 1–4. The highest scale score of 4 signifies high comfort or positive experiences, specifically eHealth literacy strengths. The lowest scale score of 1 indicates low comfort or negative experiences, specifically eHealth literacy weaknesses. The eHLQ does not provide cut‐offs or benchmarking for high or low eHealth literacy levels. A Swedish version of the eHLQ was used in this study. The original Danish eHLQ version, which has previously been validated, was translated into Swedish using the “Translation Integrity Procedure” guidelines from Deakin University to maintain measurement equivalence, while ensuring the linguistic and cultural appropriateness of the Swedish version (Hawkins et al., 2020). The accuracy of the Swedish translation was validated through face‐to‐face cognitive interviews conducted with 16 individuals across gender, age and education level.

#### 2.3.2 Socioeconomic and demographic characteristics

The following socioeconomic and demographic characteristics were measured: parents' self‐reported monthly wages (per person) (expressed in natural logarithms in the analysis for distributional purposes), educational level (secondary vs. tertiary education level), age (in years), gender (Male, Female) and residency (urban vs. semi‐urban or rural).

### 2.4 Statistical analysis

Descriptive statistics were used for socioeconomic and demographic variables. Correlations between the eHLQ domains and socioeconomic and demographic variables were assessed using Pearson (linear) correlation for continuous variables and point biserial correlation for dichotomous variables. The strength of the correlation was tested according to the threshold values of weak (≤ ± 0.2), moderate (±0.3–0.6), and strong (≥ ± 0.7) (Brace et al., 2006). All statistical analyses were performed using the Stata version 16.1. Due to the exploratory nature of the study, the alpha level of 0.10 was used (Schumm et al., 2013).

### 2.5 Ethics statement

The participants were given written information about the study and informed that participation was voluntary, and that they were able to drop out without any explanation and without any effects on usual care. Informed consent was obtained from all the participants and personal data were handled confidentially. The Swedish Ethical Review Authority (no. 2019‐0341) approved this study.

## 3 RESULTS

Of the 30 participants, in all cases, except three, parents reported Sweden as their country of birth/origin. Parents' mean age was 32.5 years (range 24–42), and males and females were evenly distributed (Table 1). Most parents reported having tertiary education (66.7%), and all others had at least secondary education (*gymnasium* in Swedish). Furthermore, most parents stated that they lived in urban areas (63%) with monthly wages per person varying between 15,000 (1,475) SEK (€) and 70,000 (6,886) SEK (€) (Table 1). All parents had children undergoing paediatric surgery, where 43% or 13 children underwent laparotomy/laparoscopic procedures, for example for appendicitis, 30% (*n* = 9) urogenital procedures, for example for renal dysplasia, 17% (*n* = 5) abdominal wall procedures, for example for inguinal hernia, and 10% (*n* = 3) cutaneous procedures, for example for abscesses.

**TABLE 1:** Parents' socioeconomic and demographic characteristics (*N* = 30)

Characteristics	*n* (%, unless otherwise stated)
**Age**	
Mean	32.53
SD	4.44
Median	32.5
Range	24–42
**Gender**	
Men	13 (43.33)
Women	17 (56.67)
**Education**	
Secondary	10 (33.33)
Tertiary	20 (66.67)
**Monthly wage**	
Mean	36,532.14
SD	11,624.66
Median	34,250 (€ 3,371)
Range	15,000 (1,475)–70,000 (6,886) SEK (€)
**Residency**	
Urban	11 (36.67)
Rural or semi‐urban	19 (63.33)

Abbreviation: SD, standard deviation.

**FIGURE 2:**
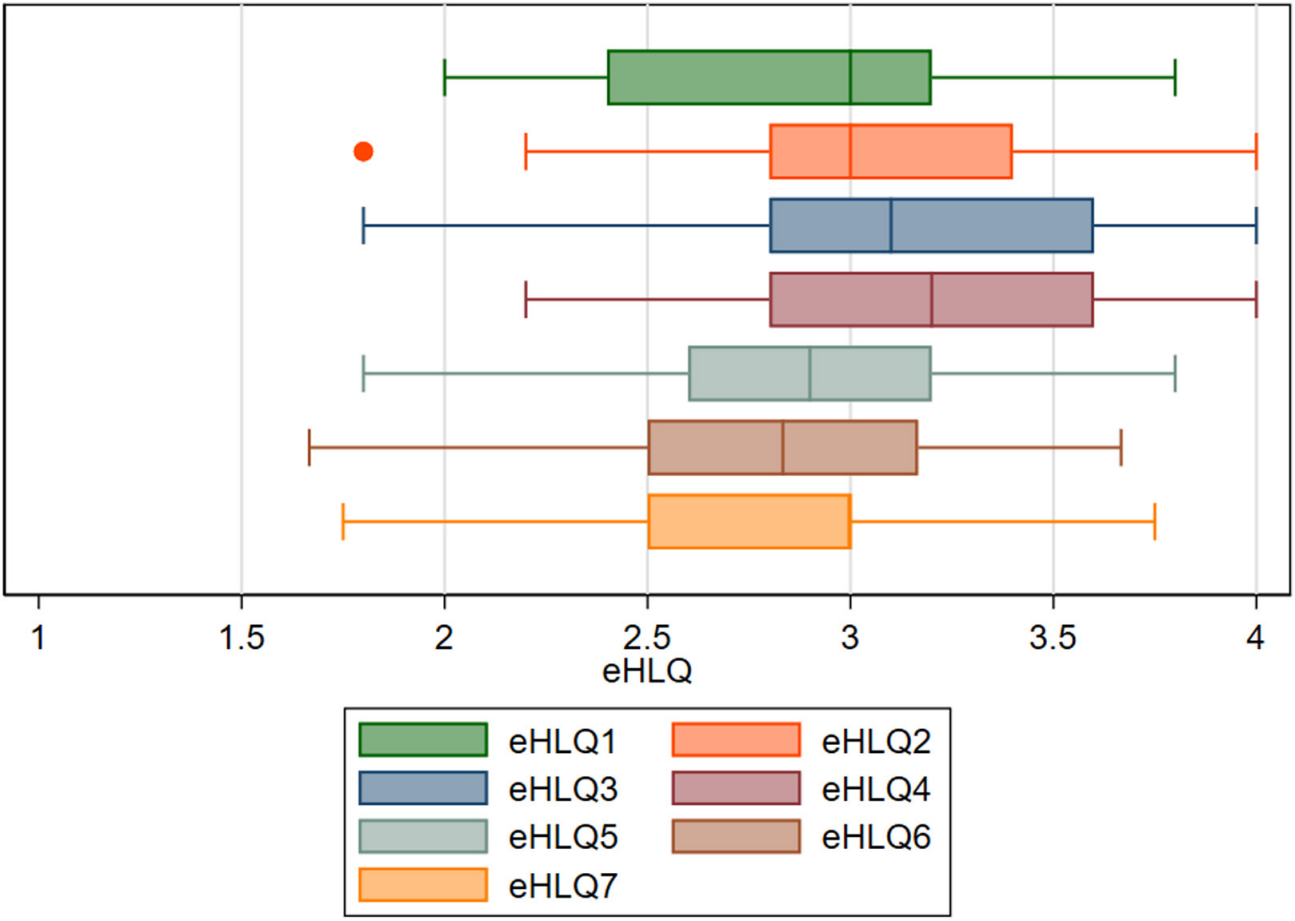
Boxplot for eHLQ1–7. The middle 50% of the observations are inside each box, with the whiskers extending 1.5 times the interquartile range left and right to the smallest and largest 25% of the observations, respectively. The adjacent lines give the upper and lower adjacent values. The dot marks an outlier value (eHLQ2). The median for eHLQ7 is 3 (not clearly visible in the boxplot). eHLQ ranges from 1 (strongly disagree) to 4 (strongly agree)

Figure 2 presents a visual overview of the spread and median scores for the seven eHLQ domains. The data shows that for most eHLQ domains, respondents' median score (the vertical bars inside the boxes) centred around “agree” (i.e. score of 3), and the highest 25% scored up to 3.5, while the lowest 25% of respondents scored down to around 2.5. The highest median scores were found on parents feeling safe and in control when interacting with digital services (eHLQ4) and the lowest with regard to their experiences of accessing digital services that work (eHLQ6). The largest spread of scores was identified on participants' ability to actively engage with digital services (eHLQ3).

Table 2 displays the means, standard deviations and range of score values for all eHLQ domains. The means were highest for domain eHLQ4: Feeling safe and in control, but lowest for domain eHLQ6: Access to digital services that work. Observed mean scores were higher on domains eHLQ1–4 (mainly representing individual attributes) than on domains eHLQ5–7 (mainly representing system attributes). Appendix A shows the sample frequency scores on each of the 35 eHLQ items, based on answers on the 4‐point scale (1 strongly disagree to 4 strongly agree).

**TABLE 2:** Descriptive statistics on the eHLQ domains

eHLQ domains	Mean	SD	Min	Max
eHLQ1: Using technology to process health information	2.92	0.52	2.00	3.80
eHLQ2: Understanding of health concepts and language	3.08	0.58	1.80	4.00
eHLQ3: Ability to actively engage with digital services	3.14	0.60	1.80	4.00
eHLQ4: Feel safe and in control	3.20	0.46	2.20	4.00
eHLQ5: Motivated to engage with digital services	2.84	0.53	1.80	3.80
eHLQ6: Access to digital services that work	2.80	0.52	1.67	3.67
eHLQ7: Digital services that suit individual needs	2.83	0.53	1.75	3.75

*Note*: The range of eHLQ is 1 (strongly disagree) to 4 (strongly agree).

Abbreviation: SD, standard deviation.

Table 3 displays the correlations of the seven eHLQ domains with socioeconomic and demographic variables. All eHLQ domains positively correlated with monthly wages. However, only parents' understanding of health concepts and language (eHLQ2) and motivation to engage with digital services (eHLQ5) correlated moderately (*r =* .329 and *r* = .347, respectively) and statistically significantly (*p =* .087 and *p* = .070, respectively) with monthly wages. Overall, high education level correlated negatively with the eHLQ domain scores, with the strongest relationship being with domain eHLQ6: Access to digital services that work, and domain eHLQ7: Digital services that suit individual needs. Both scales represent system attributes (Table 3).

**TABLE 3:** Correlation coefficients across dimensions for eHLQ and socioeconomic and demographic variables

eHLQ domains	Log monthly wage		Education		Age		Gender		Residency	
	Coefficient	*p* value	Coefficient	*p* value	Coefficient	*p* value	Coefficient	*p* value	Coefficient	*p* value
eHLQ1: Using technology to process health information	0.090	.648	−0.055	.775	−0.035	.855	0.228	.225	0.011	.955
eHLQ2: Understanding of health concepts and language	0.329	.087	−0.025	.898	0.085	.655	0.121	.523	0.182	.335
eHLQ3: Ability to actively engage with digital services	0.187	.340	−0.049	.796	−0.091	.632	−0.107	.573	0.003	.986
eHLQ4: Feel safe and in control	0.181	.357	−0.152	.421	−0.211	.263	0.087	.648	−0.149	.432
eHLQ5: Motivated to engage with digital services	0.347	.070	0.057	.766	0.032	.866	0.011	.956	0.018	.926
eHLQ6: Access to digital services that work	0.042	.831	−0.255	.175	−0.109	.566	−0.007	.971	−0.092	.629
eHLQ7: Digital services that suit individual needs	0.035	.859	−0.259	.166	−0.141	.457	−0.079	.680	−0.214	.257

*Note*: Monthly wages are expressed in natural logarithms for distributional purposes. Education is measured in two categories: secondary and tertiary education. Age is measured in years and gender and residency are categorical variables with two categories each: man/woman (reference category men) and urban/semi‐urban or rural (reference category semi‐urban or rural). Correlations are assessed using Pearson correlation for continuous variables and point biserial correlation for dichotomous variables. The strength of correlations is tested according to threshold values of Brace et al.: weak (≤ ± 0.2), moderate (±0.3–0.6), and strong (≥ ± 0.7).

Overall age correlated negatively, but weakly, with eHLQ domains. Although no relationships were statistically significant, age correlated most strongly with domain eHLQ4: Feel safe and in control (Table 3). No clear or statically significant patterns were found between gender (women vs. men as the reference category) and eHLQ domains. However, gender correlated positively and most strongly with domain eHLQ1: Using technology to process health information (Table 3). No clear or statistically significant patterns were found between residency (urban vs. semi‐urban or rural as the reference category) and eHLQ domains. However, residency correlated negatively and most strongly with domain eHLQ7: Digital services that suit individual needs (Table 3). Appendix A1, A2, A3, A4, A5 shows scatterplots for the seven eHLQ domains by the socioeconomic and demographic variables.

## 4 DISCUSSION

In this paper, we started the process of understanding different domains of eHealth literacy and their association with the socioeconomic and demographic characteristics of parents with children undergoing paediatric surgery in Sweden. To the best of our knowledge, this is the only study presenting data on this issue for this population.

Overall, self‐reported data from the 30 parents indicated adequate eHealth literacy, or what others using the eHLQ have identified as representing individuals with a “medium” eHealth literacy profile (Cheng, Elsworth, & Osborne, 2020; Kayser et al., 2019). Importantly, the mean scores and patterns on the seven eHLQ domains were congruent with other Nordic samples from clinical (Holt et al., 2019; Villadsen et al., 2020) and non‐clinical (Holt et al., 2020) settings. This suggested strengths in parents' digital competence and control (eHLQ1–4), but weaknesses with regard to feeling motivated to use digital services (eHLQ5), experiences in accessing services that work (eHLQ6), and the responsivity of systems to their needs (eHLQ7).

Globally, Sweden has a very high digital performance level (European Commission, 2020), and within digitally mature societies, individuals are expected to find digital systems accessible and responsive to their needs, especially compared with less digitally mature societies (Kayser et al., 2018). In this respect, it was surprising to find parents' eHealth literacy weaknesses centred around system performances. Nevertheless, in a recent systematic review Cheng, Beauchamp, et al. (2020) applied the eHLF to understand the role of eHealth literacy in digital intervention development among socially disadvantaged groups. The authors found that the eHealth literacy domain represented by eHLQ7: Digital services that suit individual needs, was the most overlooked domain. Whereas domains represented by eHLQ1: Using technology to process health information, and eHLQ2: Understanding of health concepts and language, were the two most frequently addressed domains (Cheng, Beauchamp, et al., 2020). These findings suggest that when developing eHealth interventions, researchers tend to pay more attention to features relating to users' digital skills, and less to features improving the performance of the digital systems, which is supported by our findings.

Therefore, based on our findings, it seems irrelevant to initiate educational programmes and practice training of digital skills among parents of children needing paediatric surgery in Sweden. Rather, moving forward, it may be more feasible to pay attention to the attributes of the digital system itself. For example, by ensuring accessibility through readily available technical support, and responsivity by empowering parents with choices tailored to their individual needs.

In this study we started the process of exploring the relationship between eHealth literacy and socioeconomic and demographic characteristics among parents of children needing advanced paediatric surgery and living in Sweden. The strongest and most consistent pattern was found between eHealth literacy and monthly wages, but there was less correlation between eHealth literacy and gender, as well as residency. As expected, our results suggest a positive association between monthly wages and eHealth literacy. This result is consistent with earlier studies (Chesser et al., 2016; Mitsutake et al., 2012). Nevertheless, these relationships were weak or moderate in strength, and given our small sample size, and the fact that no comparable studies using the eHLQ tool were found, future studies are needed to corroborate these findings.

Surprisingly, negative patterns were found between higher education and various eHealth literacy domains, although none were significant. These findings are inconsistent with the conventional literature (Holt et al., 2020; Stellefson et al., 2018), showing a positive association between these variables. However, others applying the eHLQ among Nordic older adult sample (Holt et al., 2019) have also reported statistically significant and negative relationships been education and domain eHLQ4 and eHLQ6, explaining “general skepticism toward digital services” as driving the results (Holt et al., 2019, p. 9). Our sample represents young adults, and in practice, it seems unlikely that higher education inhibits their eHealth literacy. Rather these data might derive from parents' previous experiences of working within non‐digital‐health environments who respond better to their individual needs and provide better access to technology that works, compared with the current digital health environments. As before, the dataset is too small to go deeper into assessing this relationship, but it should be further studied in future research.

Regarding gender, our findings are congruent with others which also explore the eHLQ among Nordic samples (Holt et al., 2019, 2020), suggesting weak relationships between gender and eHealth literacy. However, the literature is overall inconsistent in its reporting of gender and eHealth literacy (Huang et al., 2020; Ozen, 2021). Furthermore, our findings are in contrast with some (Paige et al., 2018), but consistent with others using the eHLQ among Nordic adults (Holt et al., 2019, 2020), in showing no strong relationship between age and eHealth literacy. Nevertheless, over the seven eHLQ domains, Holt et al. (2020) found only age to be statistically and significantly related to domain eHLQ4: Feel safe and in control. Also in our study, age related most strongly with domain eHLQ4. In fact, age is an antecedent of eHealth trust, where higher age is reflected in less trust in digital resources (Paige et al., 2017; Zulman et al., 2011). Importantly, however, as we move forward, gender and age do not establish themselves as potential barriers to eHealth literacy in this population. Yet, given the small sample size, and the lack of diversity in our sample, these relationships need further study.

This study has several limitations, the most obvious being the small sample size, and further statistical analyses requires a larger sample size to yield more statistically sound results using a significance level lower than 10% (e.g. α = 5% or lower). For example, to identify statistically significant differences for monthly wages using α = 5% and β = 20% (where β is the type 2 error and the statistical power equals 1–β), a sample size of at least 60 parents would be necessary for eHLQ2 and higher for the other domains (Rosner, 2011). Furthermore, this is a single‐site study that has limited generalizability. Additionally, participants' assessments were self‐reported and did not include more objective measures. Also, our sample is overrepresented by individuals with higher education and underrepresented by individuals in minority groups. Parents who did not read and write Swedish were not included, and therefore our results do not represent the cultural diversity of the Swedish population. Today, it is estimated that 26% of the Swedish population are of foreign background (Statistics Sweden, 2021). However, this carefully conducted study is, to our knowledge, the first to report and offer data on eHealth literacy and its associations with socioeconomic and demographic characteristics for this population of parents living in Sweden. Consequently, the present results are meaningful as they provided unique information of eHealth literacy strengths and weaknesses, or potential barriers, that may aid others in developing and implementing equitable eHealth services for these families.

eHealth disparities, or the digital divide, is an ongoing concern worldwide (Williams et al., 2020), and future analyses need to pay attention to these variables to prevent unequal healthcare. This is especially true now, given the worldwide change to virtual healthcare following the COVID‐19 pandemic (Vollbrecht et al., 2021; Webster, 2020). For this to succeed, eHealth developers are encouraged to assess and better understand end‐user diversity and eHealth literacy (Cheng, Beauchamp, et al., 2020). In future research, looking at culturally diverse groups of parents, those of children with other illnesses, or living in rural areas, could illuminate important new findings and provide more holistic views of the relationship between eHealth literacy and socioeconomic conditions in eHealth intervention development.

## 5 CONCLUSION

The results suggest that parents living in Sweden, with children undergoing advanced reconstructive paediatric surgery for gastrointestinal malformations, have adequate eHealth literacy. Although small, the dataset is unique in exploring an underrepresented population with regard to their eHealth literacy and socioeconomic and demographic characteristics. eHealth literacy barriers arose more from system attributes (access and responsivity to needs), and parents' lack of motivation to interact via current platforms, rather than from their own skills and competence using eHealth. Results suggest that information on eHealth literacy and socioeconomic conditions may aid in the development, implementation and evaluation of digital interventions. Future studies are encouraged to improve on reaching out to minority groups.

## Figures and Tables

**FIGURE A1:**
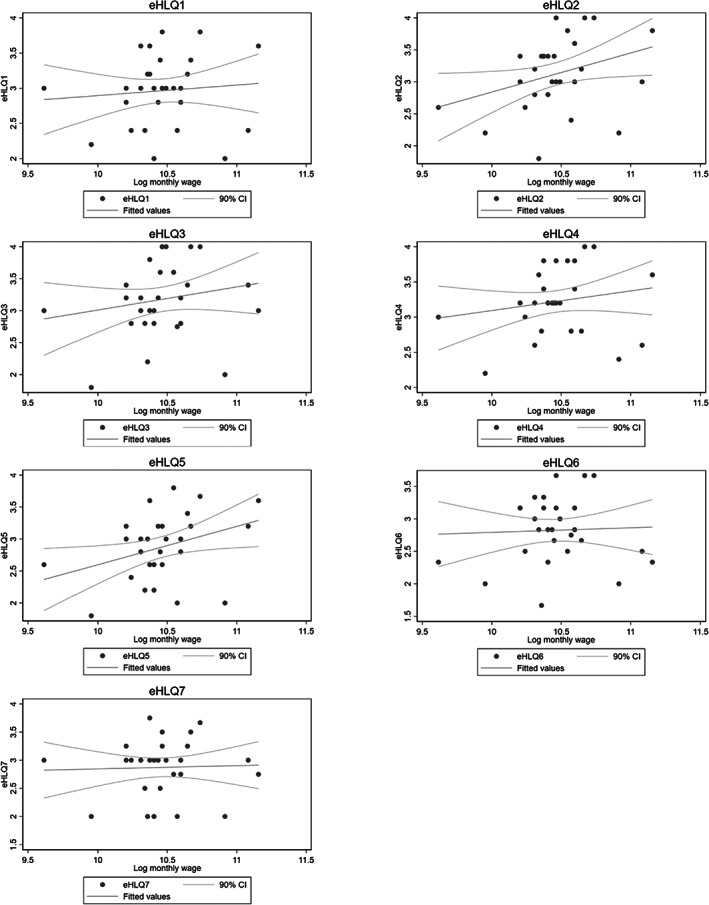
Scatterplots with fitted values using two‐variable linear regression model and 90% confidence intervals (linear model). Self‐reported monthly income (in logs) (continuous variable) on the *x*‐axes and eHLQ scores (continuous variables, range 1–4) on the *y*‐axes

**FIGURE A2:**
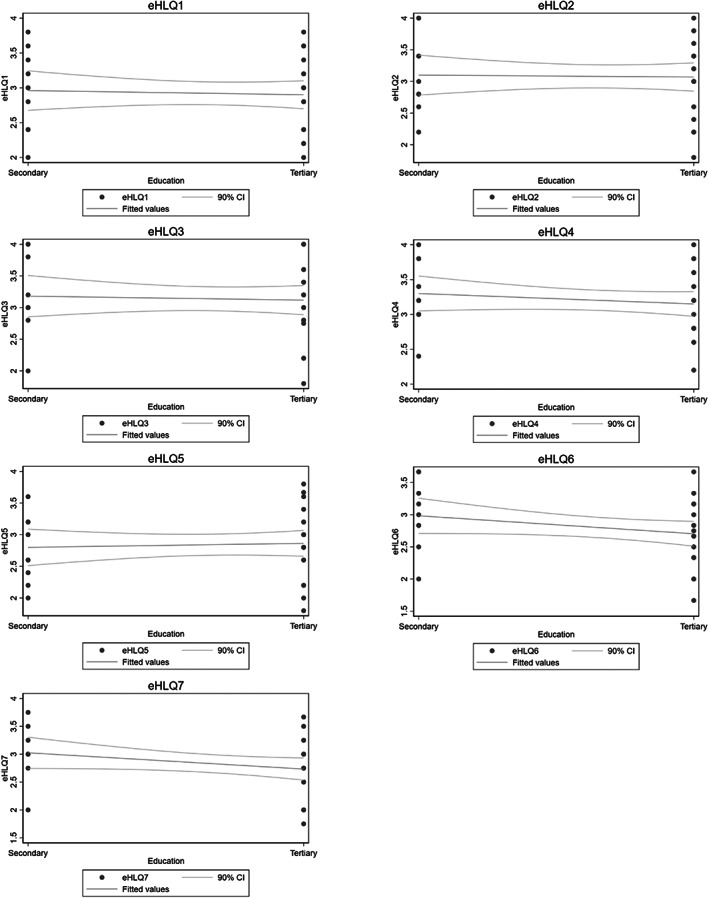
Scatterplots with fitted values using two‐variable linear regression model and 90% confidence intervals (linear model). Education status (categorical variable: secondary vs. tertiary education) on the *x*‐axes and eHLQ scores (continuous variables, range 1–4) on the *y*‐axes

**FIGURE A3:**
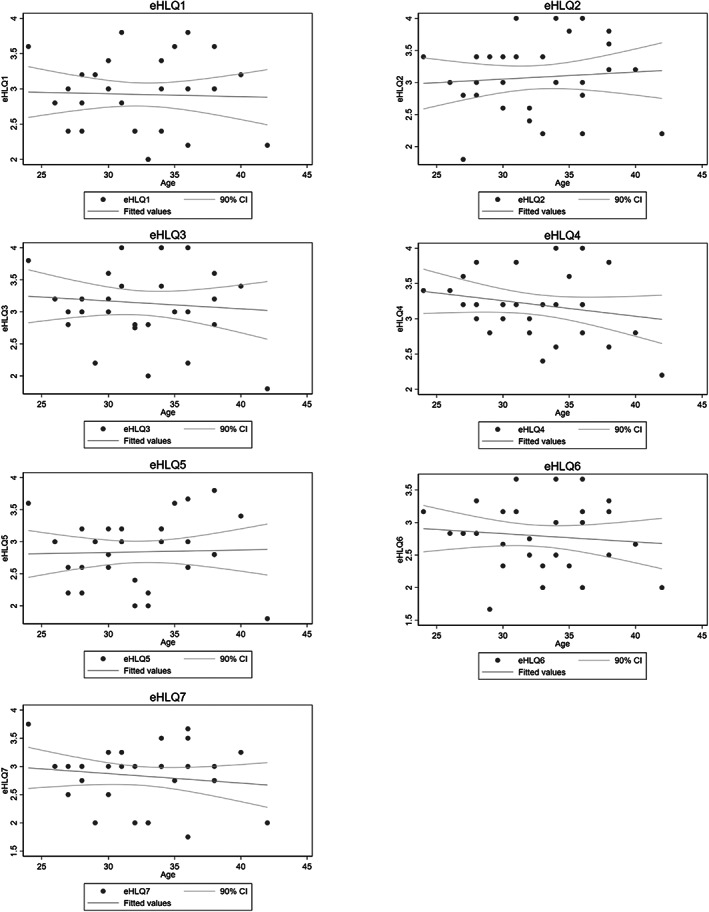
Scatterplots with fitted values using two‐variable linear regression model and 90% confidence intervals (linear model). Age (in years) (continuous variable, range 24–42) on the *x*‐axes and eHLQ scores (continuous variables, range 1–4) on the *y*‐axes

**FIGURE A4:**
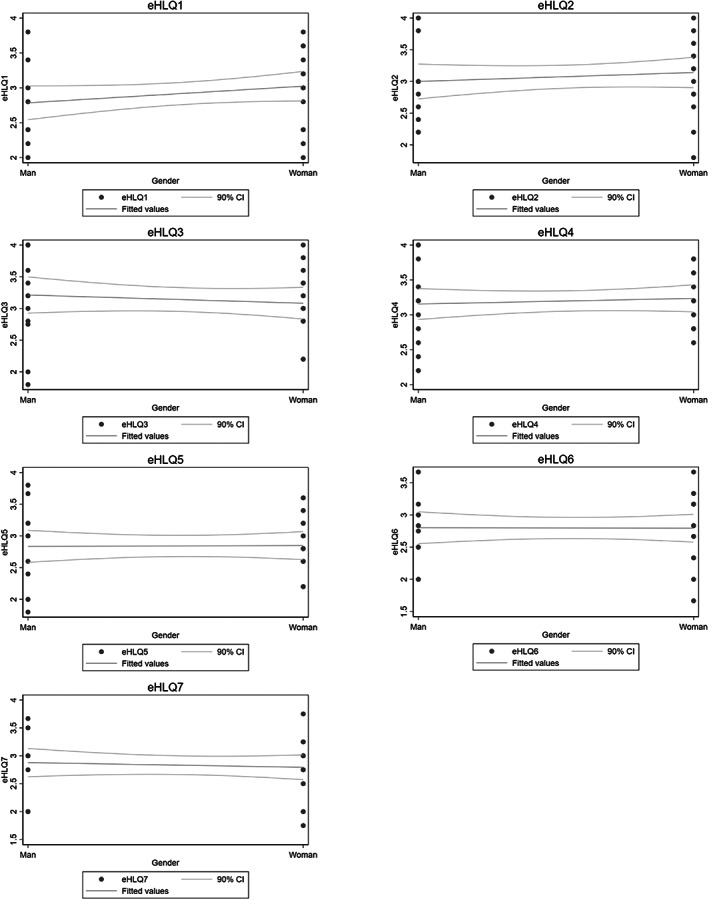
Scatterplots with fitted values using two‐variable linear regression model and 90% confidence intervals (linear model). Gender (categorical variable, men vs. women) on the *x*‐axes and eHLQ scores (continuous variables, range 1–4) on the *y*‐axes

**FIGURE A5:**
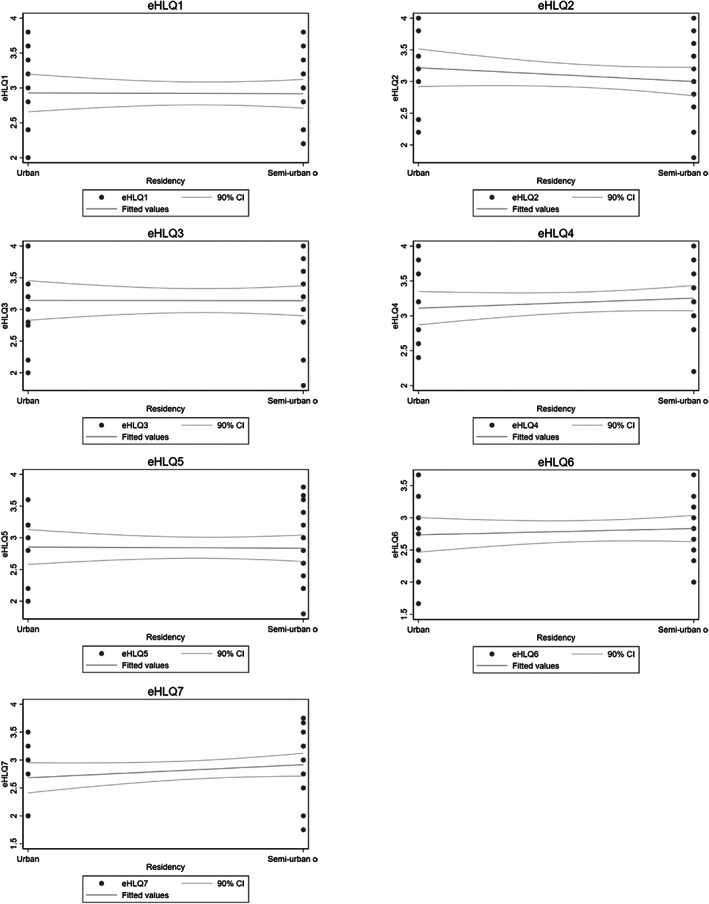
Scatterplots with fitted values using two‐variable linear regression model and 90% confidence intervals (linear model). Residency (categorical variable, urban vs. semi‐urban or rural) on the *x*‐axes and eHLQ scores (continuous variables, range 1–4) on the *y*‐axes.

## APPENDIX A

**TABLE A1:** Descriptive statistics on eHealth literacy questionnaire domains and items^a^

Domain	Item	Total number of answers (*N*)	Strongly disagree *n* (%)	Disagree *n* (%)	Agree *n* (%)	Strongly agree *n* (%)
*eHLQ1: Using technology to process health information*	I use technology to find…	30	0 (0.00)	3 (10.00)	10 (33.33)	17 (56.67)
	I often use technology to understand…	30	0 (0.00)	5 (16.67)	12 (40.00)	13 (43.33)
Median value = 3.00 Mean value = 2.92	Technology helps me decide…	30	0 (0.00)	9 (30.00)	14 (46.67)	7 (23.33)
	I use technology to share…	30	4 (13.33)	12 (40.00)	14 (46.67)	0 (0.00)
	I use technology to organize…	30	2 (6.67)	11 (36.67)	14 (46.67)	3 (10.00)
*eHLQ2: Understanding of health concepts and language*	The information I have helps me…	30	0 (0.00)	6 (20.00)	16 (53.33)	8 (26.67)
	I have enough information to take part in…	30	0 (0.00)	6 (20.00)	12 (40.00)	12 (40.00)
Median value = 3.00 Mean value = 3.08	I understand medical results…	30	0 (0.00)	5 (16.67)	17 (56.67)	8 (26.67)
	Overall, I understand how my body…	30	0 (0.00)	4 (13.33)	19 (63.33)	7 (23.33)
	I use measurements about my body to…	29	3 (10.34)	3 (10.34)	17 (58.62)	6 (20.69)
*eHLQ3: Ability to actively engage with digital services*	I know how to get…	30	1 (3.33)	4 (13.33)	14 (46.67)	11 (36.67)
	I know how to make health technology…	30	0 (0.00)	3 (10.00)	15 (50.00)	12 (40.00)
Median value = 3.10 Mean value = 3.14	I can enter data…	30	3 (10.00)	8 (26.67)	11 (36.67)	8 (26.67)
	I quickly learn how to…	30	0 (0.00)	3 (10.00)	14 (46.67)	13 (43.33)
	I easily learn to use…	29	0 (0.00)	5 (17.24)	16 (55.17)	8 (27.59)
*eHLQ4: Feel safe and in control*	I am sure that my health data…	30	0 (0.00)	0 (0.00)	12 (40.00)	18 (60.00)
	My electronic health care data are being stored…	30	0 (0.00)	3 (10.00)	19 (63.33)	8 (26.67)
Median value = 3.20 Mean value = 3.20	I have a clear understanding of…	30	0 (0.00)	8 (26.67)	15 (50.00)	7 (23.33)
	I am sure that only authorized people…	30	1 (3.33)	5 (16.67)	18 (60.00)	6 (20.00)
	I am confident that health care providers…	30	0 (0.00)	1 (3.33)	19 (63.33)	10 (33.33)
*eHLQ5: Motivated to engage with digital services*	Technology makes me feel actively…	30	2 (6.67)	6 (20.00)	11 (36.67)	11 (36.67)
	I find technology helps me…	30	0 (0.00)	7 (23.33)	18 (60.00)	5 (16.67)
Median value = 2.90 Mean value = 2.84	I find I get better services…	30	3 (10.00)	18 (60.00)	8 (26.67)	1 (3.33)
	Technology improves my communication…	29	2 (6.90)	7 (24.14)	15 (51.72)	5 (17.24)
	I find technology useful	28	0 (0.00)	2 (7.14)	18 (64.29)	8 (28.57)
*eHLQ6: Access to digital services that work*	Information about my health is always available…	30	1 (3.33)	8 (26.67)	11 (36.67)	10 (33.33)
	My health care providers deliver services that I can access…	30	2 (6.67)	7 (23.33)	16 (53.55)	5 (16.67)
Median value = 2.83 Mean value = 2.80	My health data are available…	30	1 (3.33)	5 (16.67)	15 (50.00)	9 (30.00)
	All the health technology I use works…	30	5 (16.67)	11 (36.67)	11 (36.67)	2 (6.67)
	Most of my health care providers can be accessed…	29	3 (10.34)	7 (24.14)	17 (58.62)	2 (6.90)
	I have access to health technology that…	29	0 (0.00)	5 (17.24)	21 (72.41)	3 (10.34)
*eHLQ7: Digital services that suit individual needs*	I find that digital services can adapt…	30	0 (0.00)	8 (26.67)	17 (56.67)	5 (16.67)
	I find that digital health services seem to…	29	0 (0.00)	13 (44.83)	15 (51.72)	1 (3.45)
Median value = 3.00 Mean value = 2.83	I find digital health services are provided to me in a way…	29	0 (0.00)	7 (24.14)	18 (62.07)	4 (13.79)
	Digital health services provide me with easy way…	29	1 (3.45)	5 (17.24)	17 (58.62)	6 (20.69)

^a^ Items are truncated; full list of items is available with the authors of the eHLQ. From “A multidimensional tool based on the eHealth literacy framework: development and initial validity testing of the eHealth Literacy Questionnaire (eHLQ),” by Kayser et al. (2018), *Journal of Medical Internet Research*, 20, multimedia Appendix A1, A2, A3, A4, A5. CC BY 4.0.
